# PhenoGMM: Gaussian Mixture Modeling of Cytometry Data Quantifies Changes in Microbial Community Structure

**DOI:** 10.1128/mSphere.00530-20

**Published:** 2021-02-03

**Authors:** Peter Rubbens, Ruben Props, Frederiek-Maarten Kerckhof, Nico Boon, Willem Waegeman

**Affiliations:** a KERMIT, Department of Data Analysis and Mathematical Modelling, Ghent University, Ghent, Belgium; b Center for Microbial Ecology and Technology (CMET), Ghent University, Ghent, Belgium; c Flanders Marine Institute (VLIZ), Ostend, Belgium; University of Wisconsin-Madison

**Keywords:** diversity, fingerprint, flow cytometry, machine learning, microbial communities, mixture model

## Abstract

Microbial flow cytometry can rapidly characterize the status of microbial communities. Upon measurement, large amounts of quantitative single-cell data are generated, which need to be analyzed appropriately. Cytometric fingerprinting approaches are often used for this purpose. Traditional approaches either require a manual annotation of regions of interest, do not fully consider the multivariate characteristics of the data, or result in many community-describing variables. To address these shortcomings, we propose an automated model-based fingerprinting approach based on Gaussian mixture models, which we call PhenoGMM. The method successfully quantifies changes in microbial community structure based on flow cytometry data, which can be expressed in terms of cytometric diversity. We evaluate the performance of PhenoGMM using data sets from both synthetic and natural ecosystems and compare the method with a generic binning fingerprinting approach. PhenoGMM supports the rapid and quantitative screening of microbial community structure and dynamics.

**IMPORTANCE** Microorganisms are vital components in various ecosystems on Earth. In order to investigate the microbial diversity, researchers have largely relied on the analysis of 16S rRNA gene sequences from DNA. Flow cytometry has been proposed as an alternative technology to characterize microbial community diversity and dynamics. The technology enables a fast measurement of optical properties of individual cells. So-called fingerprinting techniques are needed in order to describe microbial community diversity and dynamics based on flow cytometry data. In this work, we propose a more advanced fingerprinting strategy based on Gaussian mixture models. We evaluated our workflow on data sets from both synthetic and natural ecosystems, illustrating its general applicability for the analysis of microbial flow cytometry data. PhenoGMM supports a rapid and quantitative analysis of microbial community structure using flow cytometry.

## Abstract (precis)

Microorganisms are vital components in various ecosystems on Earth. In order to investigate the microbial diversity, researchers have largely relied on the analysis of 16S rRNA gene sequences from DNA.

## INTRODUCTION

Various tools have been developed to study and monitor microbial communities. With the emergence of 16S rRNA gene sequencing, researchers have uncovered the genotypic diversity of microbial communities to a large extent (1). However, microorganisms with the same genotype can still present different phenotypes, displaying so-called phenotypic heterogeneity (2). Therefore, instead of solely focusing on genotypic information, there is a need to combine omics data with phenotypic information (3). One such tool to study the phenotypic identity of microbial communities is flow cytometry (FCM). FCM is a high-throughput technique, measuring hundreds to thousands of individual cells in mere seconds. These measurements result in a multivariate description of each cell, derived from both scatter and fluorescence signals. The first is related to cell size and morphology, while the latter depends on either autofluorescence properties or the interaction between the cell and a specific stain.

Many algorithms exist in the field of immunophenotyping cytometry to identify separated cell populations, i.e., cells that share similar phenotypic characteristics as measured by FCM and that therefore can be grouped together. These algorithms are extensively benchmarked for different human FCM and mass cytometry data sets (4, 5). However, microbial cytometry data have a number of different characteristics. This originates from the fact that bacterial cells are typically much smaller in both cell size and volume than eukaryotic cells (6), which complicates their detection. In addition, no general antibody-based panels have been established for microbial cells due to the high complexity of microbial communities (7). One has to rely on general DNA stains, for which it is difficult to develop multicolor approaches (8). Therefore, the number of variables describing an individual bacterial cell is typically much lower than that for, for example, a human cell. As the number of bacterial taxa is much larger than the number of differentiating signals, cytometric distributions of these taxa can highly overlap (9–11). This is why automated cell population identification algorithms cannot be directly applied for the analysis of bacterial cytometry data. Consequently, data analysis pipelines should be designed to consider these characteristics.

To do so, microbiologists commonly rely on so-called cytometric fingerprinting techniques (12, 13). Such a fingerprint allows researchers to derive community-level variables in terms of the number of bins or clusters (i.e., gates), cell counts per cluster, and the position of those clusters (14), despite the fact that there are no or only a few clearly separated cell populations. The approaches that are currently used for the analysis of bacterial communities can be broadly divided into two categories: (i) manual annotation of clusters (12, 15) and (ii) automated approaches that employ binning strategies (13, 16–18). Both categories of methods have a number of drawbacks: (i) manual gating of regions of interest is laborious in time and operator dependent, (ii) traditional binning approaches result in a large number of variables (e.g., a fixed grid of dimensions 100 × 100 will result in 10,000 sample-describing variables), and because of that, (iii) only bivariate interactions of cytometry channels are considered when employing such a binning approach.

After a fingerprint has been constructed, communities are described by a contingency table that contains the abundances of groups of cells that are similar. Based on this, changes in microbial community structure can be quantified. This approach has been successfully applied to characterize dynamics of the microbiome in a multitude of environments, including pure (19, 20), synthetic (21), drinking water (22, 23), wastewater (12, 15), freshwater (24, 25), marine (16, 26), salivary (27), soil (28), and gut (29, 30) microbial communities. Cytometric fingerprint data can be summarized in what has been proposed as the cytometric or phenotypic diversity of a microbial community (13, 18). These are estimations of the diversity of a microbial community based on the cell counts per gate, bin, or cluster. If many clusters or bins contain cells, a community can be considered “rich.” If the cells are equally distributed over those clusters, a community can be considered “even.” Recent reports have shown a moderate to strong correlation between the cytometric diversity and genotypic diversity derived from 16S rRNA gene amplicon sequencing data (13, 16, 25, 30).

Our methodology makes use of Gaussian mixture models (GMMs). GMMs have been successfully applied to cytometry data before to identify separated cell populations in an automated way (31, 32). Considering microbial communities, Hyrkas et al. have shown that their GMM approach outperformed state-of-the-art immunophenotyping cytometry algorithms for the automated identification of phytoplankton populations (33). A similar approach has been recently proposed by Ludwig et al. to identify separated bacterial populations using two-dimensional cytometry data (34). By overclustering the data, GMMs can be adjusted to describe the distribution of multivariate data without the need to identify separated populations. As such, GMMs can be used as an effective fingerprinting strategy. Two additional advantages are the fact that multivariate data can be modeled at once and that the number of mixtures needed to describe the data is much lower than the number of variables resulting from traditional binning approaches.

In this work, we propose an extension of current fingerprinting approaches that we have called PhenoGMM. The methodology is able to describe the potentially many overlapping cell populations in microbial FCM data. We demonstrate that changes in community structure can be quantified based on the cytometric fingerprints derived from PhenoGMM. We evaluate its performance for synthetic and natural freshwater microbial communities and compare its performance with that of a generic binning approach. The methodology has been integrated in the R package PhenoFlow (13).

## RESULTS

### PhenoGMM correctly quantifies the community structure of *in silico* synthetic microbial communities.

In the first experiment, we evaluated the capacity of PhenoGMM to estimate the intracommunity diversity (i.e., α-diversity) of synthetic microbial communities. To this end, we simulated 400 different synthetic microbial community compositions and artificially aggregated the data of bacterial strains that were measured individually by FCM according to these compositions. Three hundred communities made up a training set; the other 100 communities made up the test set. The number of strains varied randomly between two and 20 (the total number of available strains). Community compositions were simulated using a Dirichlet distribution for three different values of the concentration parameter *a* (i.e., *a *= 0.1, 1, and 10). This parameter determines how evenly the weight is spread among the different strains. If *a* is small, only a few species are dominantly present. If *a* is large, the weight will be more evenly spread among the different strains. Its effect on the sampled proportions for *a *= 0.1, 1, and 10 is illustrated using Lorenz curves. These depict the cumulative proportion of abundance versus the cumulative proportion of bacterial species (see Fig. S1 in the supplemental material).

10.1128/mSphere.00530-20.1 FIG S1 Lorenz curves for all sampled *in silico* communities for *a* = 0.1, 1, 10. (A) Training set (300 communities); (B) test set (100 communities). Download FIG S1, PDF file, 2.1 MB. Copyright © 2021 Rubbens et al. 2021 Rubbens et al. This content is distributed under the terms of the Creative Commons Attribution 4.0 International license.

**FIG 1:**
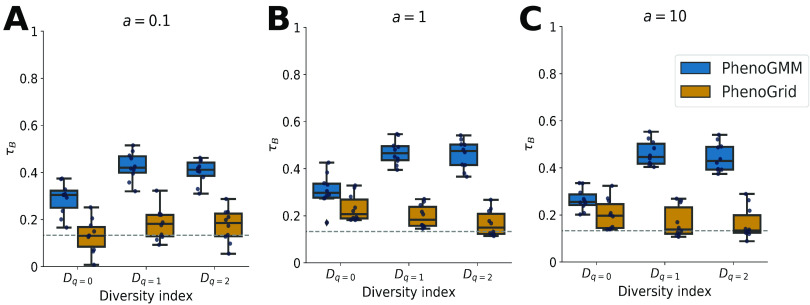
Summary of α-diversity estimations for *in silico* synthetic microbial communities, quantified by Kendall’s *τ_B_*, for PhenoGMM and PhenoGrid. Both workflows were run 10 times. Kendall’s *τ_B_* was calculated between true and estimated values. Each boxplot displays the 25% and 75% quartiles of the *τ_B_*, and the whiskers show the full range of *τ_B_*. Each dot represents the resulting value from an individual run. (A) *a *= 0.1; (B) *a *= 1; (C) *a *= 10. The dashed line indicates the strength of *τ_B_* at *P* = 0.05.

We compared PhenoGMM with an approach that we have called PhenoGrid for this work. The latter represents common cytometric fingerprinting approaches in microbial ecology that employ a binning approach to one or more bivariate combinations of the data. A GMM of *K *= 128 mixtures or a fixed binning grid of dimensions 3 × 128 × 128 (i.e., number of bivariate combinations × number of intervals first channel × number of intervals second channel) was fitted to a combined representation of the 300 communities in the training set. The resulting fingerprint templates were then used to retrieve cell counts per mixture or bin to describe each community in the test set. α-Diversity metrics were determined based on the resulting cell count contingency tables, as defined by the Hill numbers *D_q_*. The sensitivity parameter *q* determines the importance that is given to rare species or populations, with a low *q* giving more weight to rare species. α-Diversity was determined for *q *= 0 (richness), *q *= 1 (exponent of the Shannon entropy), and *q *= 2 (inverse Simpson index). Estimations of α-diversity were correlated with the “true” α-diversity values, which were based on the predefined compositions with which the communities in the training and test sets were simulated. Correlations were quantified using Kendall’s rank correlation coefficient τ*_B_* and summarized in Fig. 1. PhenoGMM resulted in moderate to highly correlated α-diversity estimations and showed a better correspondence to the predefined community compositions compared to PhenoGrid. Estimations were just above the significance level (*P* = 0.05) for the latter. The performance mainly depended on the sensitivity parameter *q*. Estimations resulted in higher correlations for PhenoGMM when *q *> 0, i.e., when more weight was given to more abundant strains. This means that PhenoGMM captured the structure rather than the identity of a microbial community. This effect was less clear for PhenoGrid.

**FIG 2:**
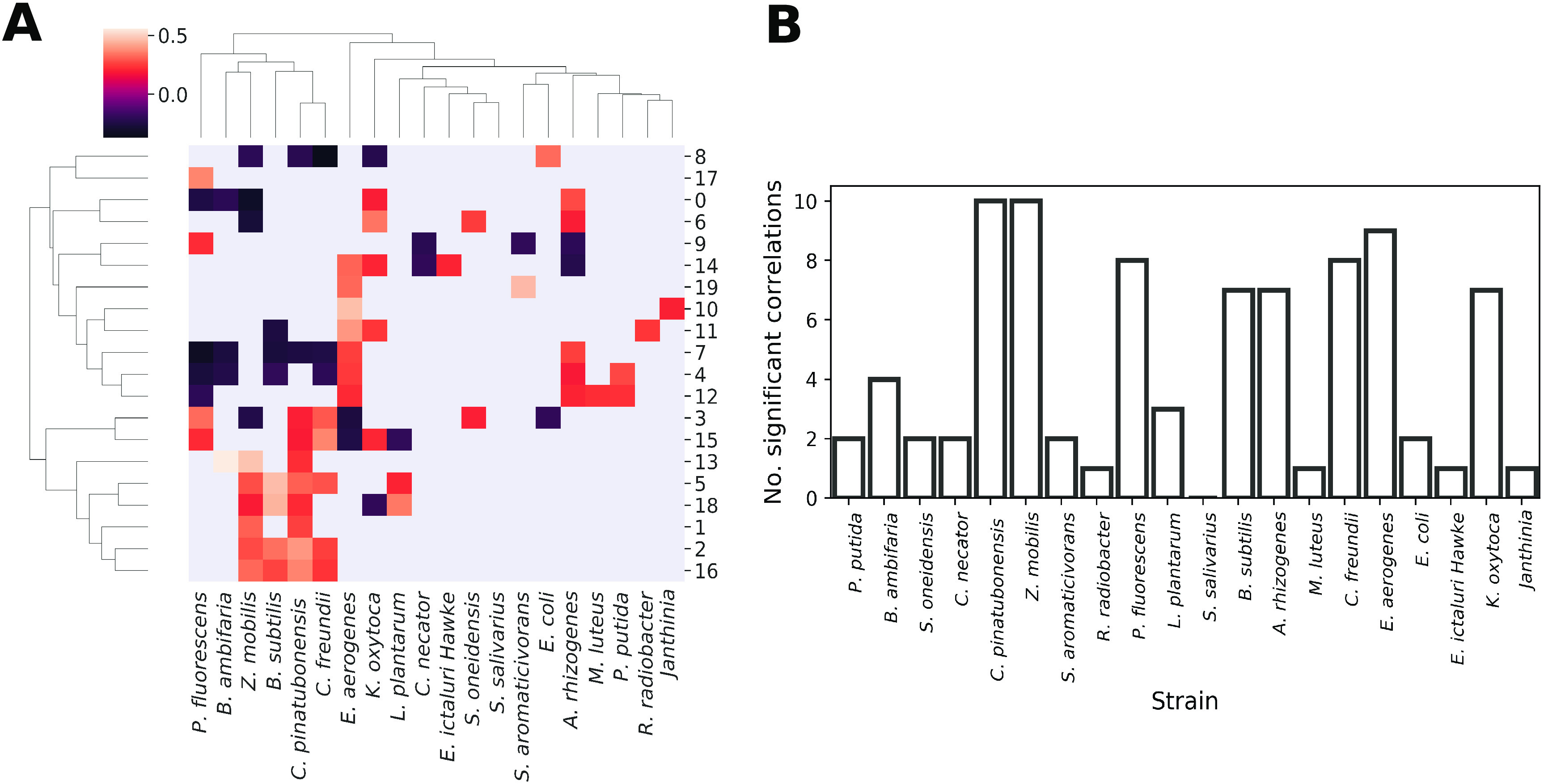
Summary of the correspondence between individual bacterial strains and the cell counts for each Gaussian mixture. (A) Kendall’s *τ_B_* between relative cell counts per mixture (rows) and relative abundances of bacterial strains (columns). Values are given if *P* is ≤0.05, after performing a Benjamini-Hochberg correction for multiple hypothesis testing. (B) Number of significant correlations per bacterial strain.

We further evaluated to what extent a mixture corresponded to one or more bacterial strains. To do so, we constructed a fingerprint using 20 mixtures for the setting in which the concentration parameter of the Dirichlet distribution was set to *a *= 1. Relative cell counts per mixture were correlated with variations in individual abundances of bacterial strains (Fig. 2A). In most cases multiple mixtures were correlated with multiple strains (Fig. 2B), which could be explained by the fact that the cytometric characterizations of the considered bacterial strains overlapped in various degrees. At the same time, no mixture was correlated with all bacterial strains, demonstrating that despite the overlapping structure in a cytometric fingerprint, variations in the mixtures could be successfully related to variations in individual strains.

**FIG 3:**
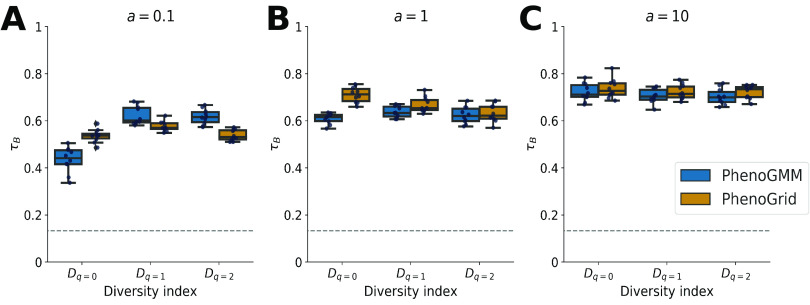
Summary of Random Forest predictions of α-diversity for *in silico* synthetic microbial communities, quantified by Kendall’s *τ_B_*, for PhenoGMM and PhenoGrid. Both workflows were run 10 times. Kendall’s *τ_B_* was calculated between true and estimated values. Each boxplot displays the 25% and 75% quartiles of the *τ_B_*, and the whiskers show the full range of *τ_B_*. Each dot represents the resulting value from an individual run. (A) *a *= 0.1; (B) *a *= 1; (C) *a *= 10. The dashed line indicates the strength of *τ_B_* at *P* = 0.05.

To scrutinize the results and potentially facilitate future synthetic microcosm experiments, we performed a predictive modeling analysis. Cytometric fingerprints from PhenoGMM and PhenoGrid were fed to a Random Forest model in order to predict the community structure according to which microbial communities were assembled in the test set. Cytometric fingerprints from both approaches either resulted in comparable predictions according to Kendall’s τ*_B_* (Fig. 3) or were slightly in favor of PhenoGMM according to *R*^2^ (Fig. S2). Random Forest predictions resulted in stronger correlations with the simulated community compositions compared to directly applying the Hill numbers to the relative cell contingency table.

10.1128/mSphere.00530-20.2 FIG S2 Summary of predictions of α-diversity for *in silico* synthetic microbial communities, quantified by *R*^2^, using PhenoGMM and PhenoGrid. PhenoGMM and PhenoGrid were run 10 times. The *R*^2^ was calculated between true and estimated values. Each boxplot displays the 25% and 75% quartiles of the *τ_B_*, and the whiskers show the full range of *R*^2^. (A) *a *= 0.1; (B) *a *= 1; (C) *a *= 10. Download FIG S2, PDF file, 0.4 MB. Copyright © 2021 Rubbens et al. 2021 Rubbens et al. This content is distributed under the terms of the Creative Commons Attribution 4.0 International license.

We estimated the time to run PhenoGMM for *a *= 1 and *D_q_*_=1_ in function of the number of mixtures *K*. As there were 300 samples in our training set, this amounted to fitting a GMM to 1.5 million cells. The time in seconds was determined in function of *K* (Fig. S3A). Most importantly, the entire analysis remained under 1 h. Training a Random Forest model on the fitted GMM resulted in an average increase of 24.4% of the runtime for *K *= 256 (Fig. S3B).

10.1128/mSphere.00530-20.3 FIG S3 Benchmarking of PhenoGMM in function of the time in seconds. Each analysis was run on a separate node of a computer infrastructure, with 2.6 GHz CPU and 20 GB of RAM for each node. (A) Time to fit a GMM and perform estimations of cytometric diversity for *D_q_*_=1_ (blue line), evaluated by τ*_B_*(*D_q_*_=1_) (orange line). (B) Time to fit a GMM, fit a Random Forest regression model, and perform predictions of α-diversity for *D_q_*_=1_ (blue line), evaluated by τ*_B_*(*D_q_*_=1_) (orange line). PhenoGMM was run five times for different *K*, for which the mean and corresponding standard deviation are visualized. Download FIG S3, PDF file, 0.5 MB. Copyright © 2021 Rubbens et al. 2021 Rubbens et al. This content is distributed under the terms of the Creative Commons Attribution 4.0 International license.

In order to provide guidance concerning use of the model, the most important parameters were varied one by one (i.e., the number of included detectors *D*, the number of mixtures *K*, the number of cells sampled per file to fit a GMM denoted as N_CELLS_MIN, the number of cells sampled per individual sample to determine the cell counts per mixture denoted as N_CELLS_REP, a learning curve in function of N_SAMPLES, and the TYPE of covariance matrix used to fit a GMM). The performance was quantified using *R*^2^ (*D_q_*_=1_), based on the communities for a concentration parameter of *a *= 1, for the same Random Forest analysis as described above (Fig. S4). The results indicated that:

• Including additional detectors improved the performance.
• Generally, the higher the number of mixtures *K*, the better the performance, which saturated after a specific threshold.
• PhenoGMM was quite robust for the number of included cells to fit a GMM.
• PhenoGMM was quite robust for the number of included cells per sample.
• The predictive performance did not saturate yet at after 300 samples.
• PhenoGMM was quite robust for the type of used covariance matrix, although the “full” type (i.e., each mixture has its own covariance matrix) resulted in the best predictions.

10.1128/mSphere.00530-20.4 FIG S4 Influence of different parameters within PhenoGMM on predictions of *D_q_*_=1_ for *in silico* communities sampled with a concentration parameter of *a *= 1. The quality of predictions is quantified by the *R*^2^. Declaration of parameters: *D*, number of included detectors (height-signal); *K*, number of mixtures; N_CELLS_MIN, number of cells that are sampled per community and concatenated (c) to fit a GMM; N_CELLS_REP, number of cells that are sampled per individual community to derive cell counts using a fitted GMM; TYPE, type of GMM that is used. Download FIG S4, PDF file, 2.2 MB. Copyright © 2021 Rubbens et al. 2021 Rubbens et al. This content is distributed under the terms of the Creative Commons Attribution 4.0 International license.

### PhenoGMM retrieves the community structure of natural freshwater microbial communities.

**FIG 4:**
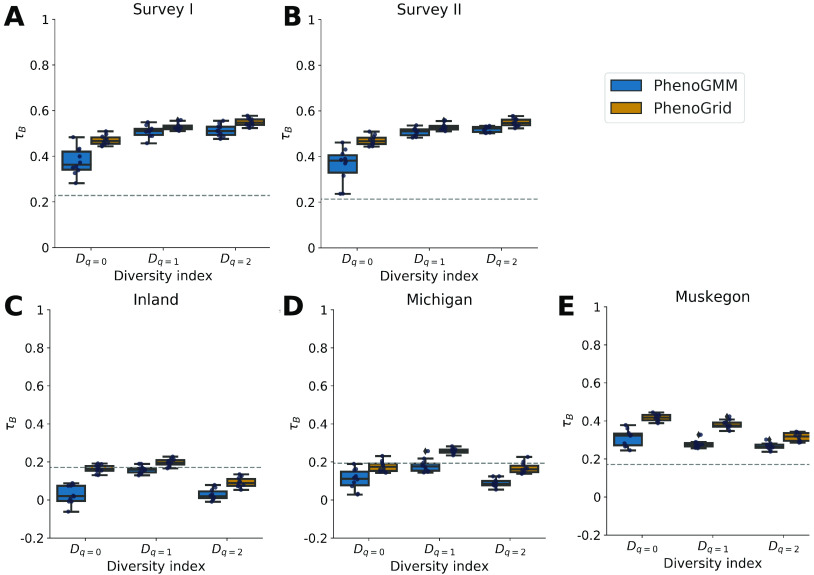
Summary of α-diversity estimations for the cooling water and freshwater lake microbiomes, evaluated by Kendall’s *τ_B_*, using PhenoGMM and PhenoGrid. Both methods were run 10 times. Kendall’s *τ_B_* was calculated between true and estimated diversity values. Each boxplot displays the 25% and 75% quartiles of the *τ_B_*, and the whiskers show the full range of *τ_B_*. Each dot represents the resulting value from an individual run. (A and B) Results for the cooling water microbiome. (A) Survey I. (B) Survey II. (C to E) Results for the freshwater lake microbiome. (C) Inland lakes. (D) Lake Michigan. (E) Muskegon Lake. The dashed line indicates the strength of *τ_B_* at *P* = 0.05.

In the second experiment, we evaluated whether and to what extent it was possible to quantify the diversity of natural freshwater microbial communities using FCM in combination with PhenoGMM. We used two data sets, of which the first describes the dynamics of a cooling water microbiome during two surveys of a research nuclear reactor (surveys I and II) and the second describes the microbiomes of three different freshwater lake systems (i.e., Michigan inland lakes [“Inland”], Lake Michigan, and Muskegon Lake, respectively). The same approach as before was applied, and PhenoGMM and PhenoGrid were compared. Samples were first aggregated to determine a fingerprint template based on either a GMM or a gridded binning approach. Next, cell counts per mixture or bin were retrieved per sample, based on which the α-diversity values were calculated. To estimate how well both methods were able to retrieve the taxonomic structure of the microbial community, these values were compared with α-diversity estimations based on 16S rRNA gene amplicon sequencing. The correspondence was again evaluated using Kendall’s τ*_B_* and summarized in Fig. 4.

Diversity estimations were highly significant for the cooling water microbiome for both approaches. The α-diversity of the microbial communities in Muskegon Lake could be successfully retrieved as well. For *q *= 1 (the exponent of the Shannon entropy), estimations were significant based on PhenoGrid, but not for PhenoGMM. In most cases, PhenoGrid outperformed PhenoGMM, indicating that more mixtures or additional detectors might be needed to make it competitive with PhenoGrid in this setting. To summarize, PhenoGMM successfully quantified the community structure of most considered natural communities, but its ability depended on the ecosystem of study and its specific implementation. In the current implementation, PhenoGrid seems to be favored, with small to moderate differences between the two approaches.

### PhenoGMM quantifies intercommunity differences.

**FIG 5:**
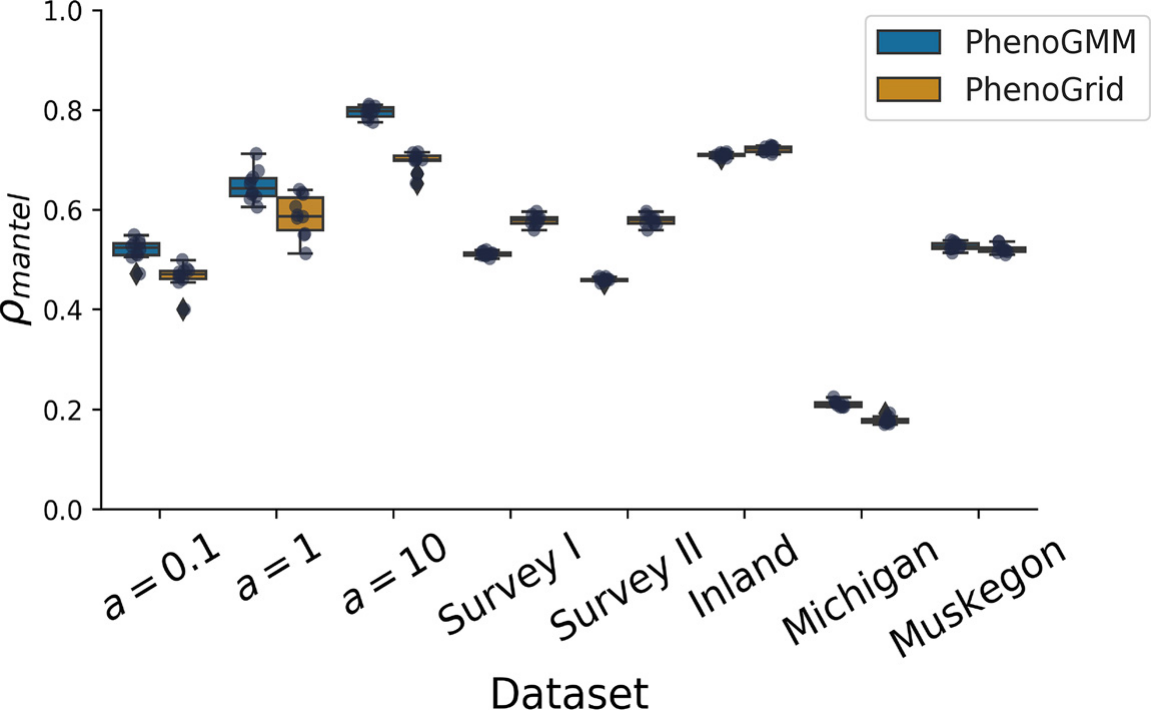
Summary of *β*-diversity estimations for all data sets, evaluated by *ρ*_mantel_, for both PhenoGMM and PhenoGrid. Both methods were run 10 times. *ρ*_mantel_ was calculated between the Bray-Curtis dissimilarity matrices based on cytometric fingerprints and the simulated synthetic community composition or 16S rRNA gene amplicon sequencing (cooling water and freshwater lake communities). Each boxplot displays the 25% and 75% quartiles of *ρ*_mantel_, and the whiskers show the full range of *ρ*_mantel_.

We also evaluated the possibility to quantify intercommunity diversity (i.e., β-diversity) for both approaches. We used the Bray-Curtis dissimilarity to quantify these differences based on the resulting cell contingency tables for each data set. A Mantel test was used to calculate the correlation between the dissimilarity matrix based on the cytometric fingerprints and the one derived from the *in silico* synthetic microbial community composition or the composition based on 16S rRNA gene sequencing for the cooling water and freshwater lake data sets. This was done for PhenoGMM and PhenoGrid (Fig. 5). Both approaches resulted in strong correlations (*P* < 0.001 for all considered communities, except Lake Michigan, for which *P* < 0.05). PhenoGMM-based fingerprints resulted in higher correlations for the simulated synthetic microbial communities, Lake Michigan and Muskegon Lake, while PhenoGrid-based fingerprints resulted in higher correlations for the cooling water and inland lake system microbiome.

## DISCUSSION

In this paper we propose a data-driven cytometric fingerprinting strategy based on Gaussian mixture models (GMMs), which we have called PhenoGMM. Our approach allows the derivation of information-rich variables from microbial cytometry data in order to describe the community structure. One of its advantages is that the method reduces the number of community-describing variables considerably compared to traditional binning approaches. We evaluated the performance of PhenoGMM in terms of the α-diversity, as quantified by the Hill numbers *D_q_* for *q *= 0, 1, and 2. These are the equivalents of the richness, exponent of the Shannon entropy, and inverse Simpson index. We also evaluated to what extent intercommunity differences can be quantified to perform *β*-diversity estimations. Both synthetic and natural microbial communities were considered. We compared PhenoGMM with the performance of a generic traditional binning approach that is representative for common approaches for cytometry fingerprinting in microbial ecology, which we have called PhenoGrid for this work.

In the first part of the paper, we constructed synthetic microbial communities *in silico* by aggregating cytometric characterizations of individual bacterial strains according to predefined compositions. This allowed us to simulate microbial community compositions in a highly precise and controlled way. These predefined compositions were used to calculate α- and *β*-diversity values. Cytometric diversity, based on the resulting cell counts for PhenoGMM and PhenoGrid, was benchmarked with the predefined values, by calculating Kendall’s rank correlation coefficient τ*_B_* between the two sets of values. Both approaches resulted in moderate to strong correlations. PhenoGMM resulted in stronger or equally accurate estimations compared to PhenoGrid. The exponent of the Shannon entropy (*D_q_*_=1_) and the inverse Simpson index (*D_q_*_=2_) were better estimated compared to the richness of the community, indicating that community structure rather than identity is captured by the fingerprints. The total analysis time of PhenoGMM remained under 1 h for the analysis of 1.5 million cells.

In the second part, we evaluated to what extent PhenoGMM was able to retrieve the structure of natural communities. Two types of ecosystems were considered, the cooling water microbiome during two surveys of a research nuclear reactor and the microbiome of three freshwater lake systems. Correlations with the taxonomic diversity based on 16S rRNA gene amplicon sequencing data have been demonstrated in previous work (13, 25) and were therefore used as a benchmark. Depending on the ecosystem of study, correlations of different strengths were observed. Considering the cooling water microbiome, moderate to strong correlations were reported for both surveys. Differences between PhenoGMM and PhenoGrid were small, but results were in favor of PhenoGrid. When considering freshwater lakes, only the data from Muskegon Lake resulted in significant correlations for PhenoGrid. The exponent of the Shannon entropy resulted in significant correlations as well for the other two lake systems, indicating again that cytometric fingerprinting approaches capture community structure rather than identity.

Note that we do not expect to find a “perfect” correlation between the cytometric and taxonomic diversity. Besides the fact that 16S rRNA gene amplicon sequencing is subject to a number of biases (35, 36), microbial FCM is sensitive to both taxonomic and physiological changes. Therefore, the strength of the correspondence between cytometric and taxonomic diversity will vary from experiment to experiment and from system to system, with multiple factors affecting the strength of the correspondence. First, the freshwater lake microbiome displays larger values in richness and evenness compared to the cooling water microbiome (25). Second, the levels of trophicity differ between the considered data sets, which could be affecting the estimations. Third, the sampling coverage is different between the data sets. The cooling water microbiome contains many measurements over a few days in a highly dynamic system, compared to the freshwater lake microbiome that contains samples spanning a much larger range in time (years) and space (multiple locations).

Estimations of *β*-diversity (i.e., intercommunity diversity) could be successfully quantified as well, by calculating Bray-Curtis dissimilarities between the cytometric fingerprints of different communities. A Mantel test demonstrated that correlations were significant for all data sets and strong in most cases, indicating that in some cases it could be more worthwhile to investigate inter- rather than intracommunity differences.

Few reports exist that quantitatively evaluate fingerprinting approaches for the analysis of microbial cytometry data. Most fingerprinting strategies make use of manual annotation of clusters or of fixed binning approaches (see, e.g., the report by Koch et al. [14] which qualitatively discusses different existing methods). In almost all cases, only bivariate interactions are inspected. PhenoGMM allows modeling the full parameter space at once. This is interesting, because although it is difficult to develop multicolor approaches for bacterial analyses, these are possible (see, e.g., the work by Barbesti et al. [37]). In addition, our research group has demonstrated that additional detectors that capture signals due to spillover can assist in the discrimination between bacterial species (38). Therefore, the parameter space in which bacterial cells can be described is increasing, and PhenoGMM is able to model this straightforwardly. Because it is an adaptive strategy as well, by defining small clusters in regions of high density and vice versa, it reduces the number of sample-describing variables considerably compared to fixed binning approaches. In that sense, it shares some properties with FlowFP. This is an adaptive binning approach, in which bins are smaller when the density of the data is higher and vice versa. However, the bins are still hyperrectangular in shape, while PhenoGMM allows clusters to be of any shape. Other adaptive binning strategies have been proposed for microbial FCM data as well (24); however, these are still limited to bivariate interactions.

Our approach comes with a number of caveats. First, PhenoGMM fits a fingerprint template based on the concatenation of measured samples. New samples are characterized based on this template. In the case that multiple samples diverge considerably from those that were used to determine the template (for example, in the case that an experiment was conducted under different conditions), we recommend refitting the model. Second, we overcluster the data to model the multiple and potentially overlapping cell distributions due to the differences in physiology and the many species that can be present in a microbial community. This makes it difficult to determine the exact number of mixtures. As the number of mixtures *K* increases, the performance saturates gradually, and more mixtures will not improve estimations. Therefore, *K* should be chosen high enough but might differ from experiment to experiment. PhenoGMM can also be tailored toward the identification of separated cell populations, for example, to identify phytoplankton populations (33), for the identification of so-called high- and low-nucleic-acid groups (39), or to identify distinct bacterial populations when the resolution of the data is high enough (34). In this case, if the number of populations is known beforehand, *K* can be chosen accordingly; if this is not known, one can use decision rules such as the Bayesian information criterion (BIC) to determine the optimal number of mixtures (34). Third, due to overclustering of the data, mixtures can be highly correlated with each other.

Our *in silico* benchmark study made use of cytometric characterizations of individual bacterial strains. Individual cultures are known to exhibit considerable heterogeneity due to cell size diversity and cell cycle variations (40). Our research group has recently shown that the cytometric diversity of an individual culture reduces when it is part of a coculture (21). Therefore, data used for the *in silico* community creation setup cannot be used to study environmental samples, as we hypothesize that members of natural communities will have a different cytometric fingerprint from strains that were grown and measured individually. Yet, we believe that our *in silico* community assembly approach is useful, as it allows a precise simulation of variations in cytometric community structure. 

To conclude, PhenoGMM can be used to derive information-rich variables from microbial FCM measurements. Microbial community structure can be quantified by computing cytometric diversity metrics based on the PhenoGMM-based fingerprints. The method has a number of advantages compared to traditional cytometric fingerprinting approaches. To facilitate its use by the scientific community, it has been integrated in the R package PhenoFlow (13). Technological advancements have enabled an automated data acquisition, resulting in a detailed characterization of the microbial community online (i.e., samples are measured at routine intervals between 5 and 15 min) or in real time (i.e., near-continuous measurements) (41, 42). Therefore, we see great potential to use FCM as a monitoring technique to rapidly and frequently investigate microbial community dynamics, which can be supported by PhenoGMM. It has to be noted that quantification of diversity should serve as a starting point to test ecological hypotheses rather than as a final outcome of an experiment (43). Microbial FCM, in combination with PhenoGMM, has the potential to be an effective strategy to serve this research line in microbial ecology.

## MATERIALS AND METHODS

### Methodology.

In this work, multiple data sets were analyzed. Each data set contained multiple FCM samples, either individual bacterial strains or natural communities. Bacterial cells were described by scatter and fluorescence signals, for which the latter resulted from the use of a nucleic acid stain (SYBR green I). Experimental details per data set are laid out in detail below. We first describe the methodology of PhenoGMM.

### Preprocessing.

Two preprocessing steps are applied to all cytometry samples before further analysis of the data. First, all individual FCM channels are transformed by *f*(*x*) = asinh(*x*). Next, background due to debris and noise is removed using a fixed digital gating strategy (13, 44). In other words, a single gate is applied to separate bacterial cells from background and is used for all samples. This gate is fixed within a specific experiment but can differ from data set to data set.

### Cytometric fingerprinting using Gaussian mixture models.

When the preprocessing is completed, a fingerprint template or model needs to be determined that is able to describe all the samples within an experiment. Therefore, samples are first subsampled to the same number of cells per sample (N_CELLS_MIN), in order to not bias the Gaussian mixture model (GMM) toward a specific sample, and concatenated in a training set. This number can be either the lowest number of cells present in one sample or a number of choice. A rough guideline can be to not let the training set be larger than 1 × 10^6^ cells, but this depends on computational resources. If *n* denotes the total number of samples, then the total number of cells (N_CELLS) in the training set will be determined as N_CELLS = *n* × N_REP × N_CELLS_MIN, in which N_REP denotes the number of technical replicates of a specific sample. Typically, forward (FSC) and side (SSC) scatter channels are included, along with one or more targeted fluorescence channels (denoted as FL*X*, in which *X* indicates the number of a specific fluorescence detector). Unless noted otherwise, channels FSC-H, SSC-H, and FL1-H (530/30 nm) are included for data analysis.

Once this training set is created, a GMM of *K* mixtures is fitted to the data. If **X** denotes the entire data matrix or training set containing *N* cells, then **X** consists of cells written as ***x***_1_, …, ***x****_N_*, of which each cell is described by *D* variables (i.e., the number of signals collected from the flow cytometer). Cell *i* is described as $x_{i}=\{x_{i1},\ldots ,x_{iD}\}$. A GMM consists of a superposition of normal distributions *N*, of which each distribution has its own mean μ and covariance matrix Σ. Each mixture has a mixing coefficient or weight π, which represents the fraction of data each mixture is describing. The distribution *p* can be written as follows:

(1) $$p(X)=\sum_{k=1}^{K}{\text{π}}_{k}N(X|\mu_{k},\Sigma_{k}).$$

**FIG 6:**
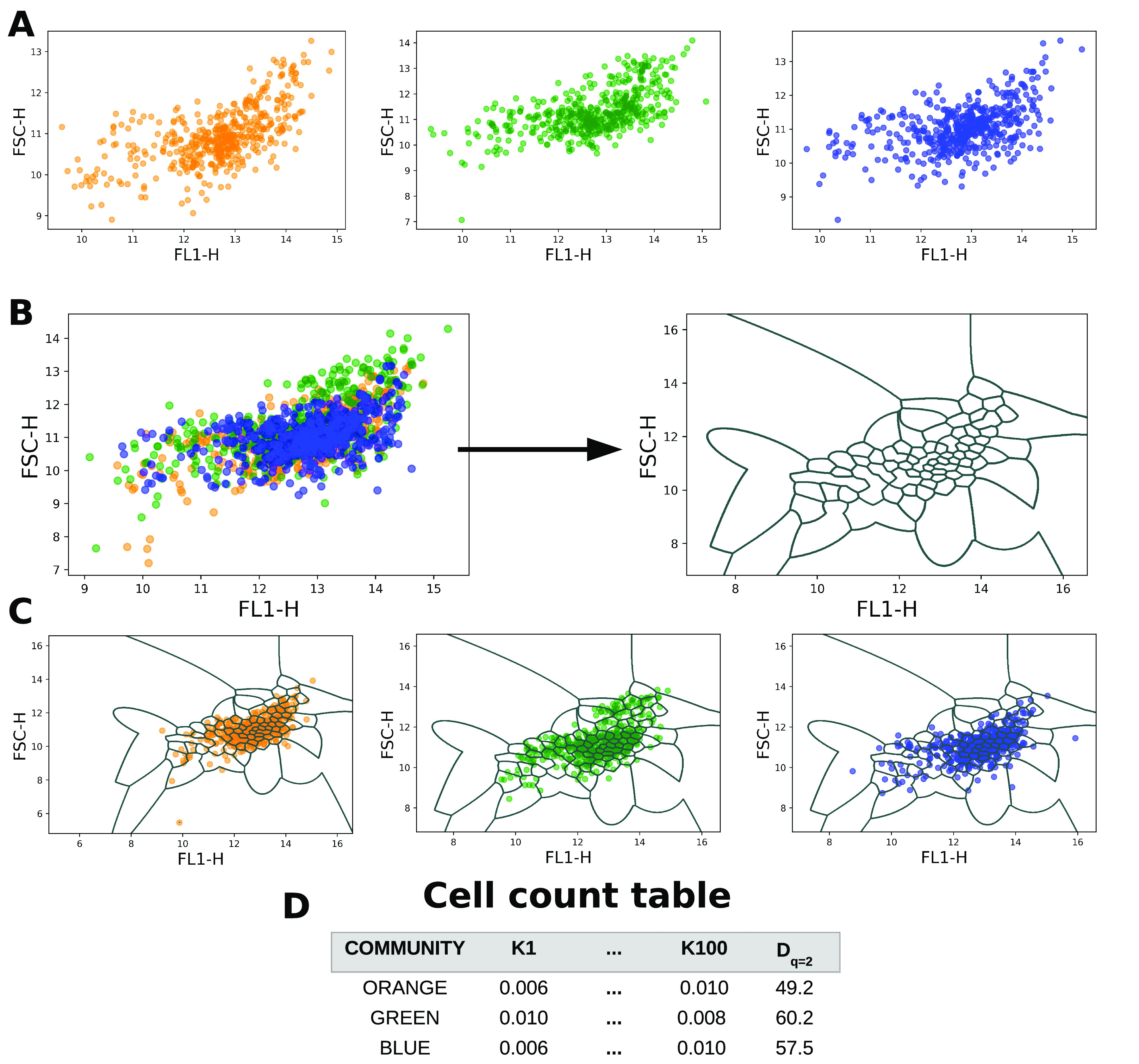
Illustration of PhenoGMM for two channels (FL1-H and FSC-H) using *K *= 100 mixtures. (A) The analysis starts from cytometric measurements of three bacterial communities of interest, noted as “ORANGE” (*S *= 6), “GREEN” (*S *= 8), and “BLUE” (*S *= 15). (B) Data for the three communities are concatenated into one data frame, to which a GMM with (in this case) *K *= 100 mixtures is fitted. This results in a fingerprint template, which is depicted on the right. (C) The fingerprint template is used to derive relative cell counts per cluster and per bacterial community. (D) This results in a “count” table, which can be used to rapidly quantify the cytometric diversity based on equations 2 to 4 (in this case *D*_2_).

The set of parameters $\Theta ={\left[\pi_{k},\mu_{k},\Sigma_{k}\right]}_{k=1}^{K}$ is estimated by the expectation-maximization (EM) algorithm (45). Once a GMM has been trained on the concatenated data, the fingerprint template is determined and one can assign all cells per sample to the mixture for which it has the highest posterior probability. This time, replicate samples are subsampled to a specific number of cells or the lowest number of cells of the replicates that are part of that specific sample and pooled. This number is denoted as N_CELLS_REP. After clustering, the number of cells per mixture is counted, after which the relative number of cells per mixture and sample is retrieved, further defined as the cytometric fingerprints. An illustration of PhenoGMM can be seen in Fig. 6.

We used the GaussianMixture() function of the scikit-learn machine learning library to implement our method (46). This function contains four different ways to estimate the covariance matrix of each mixture:

• diag: each mixture has its own diagonal covariance matrix.
• full: each mixture has its own general covariance matrix.
• spherical: each mixture has its own single variance. (Note this is not the same as *k*-means clustering. In this case, all mixtures would share the same single variance.)
• tied: all mixtures share the same general covariance matrix.

Unless otherwise noted, we let each mixture have its own general covariance matrix (full). mClust was used to integrate PhenoGMM in the R package PhenoFlow (47).

### Defining cytometric diversity.

Cytometric fingerprints allow definition of the cytometric diversity of a microbial community (13, 18). If one considers each predefined gate or mixture as a phenotypic unit, one can calculate both intra- and intercommunity diversity metrics, also known as α- and *β*-diversity. The first quantifies the diversity within a sample, and the latter quantifies the diversity between samples. Various diversity metrics exist in ecology to calculate α-diversity. In this work, we apply the Hill numbers *D_q_*(**p**) to quantify community diversity (48), as proposed by Leinster and Cobbold (49) and Daly et al. (50). If we let **p** = *p*_1_, …, *p_S_* represent the vector of relative abundances, describing the abundance of *S* bacterial species or populations, then we can define the richness (*D_q_*_=0_) and evenness (*D_q_*_=1_, *D_q_*_=2_) of a microbial community as follows:

(2) $$D_{q=0}(p)=S,$$

(3) $$D_{q=1}(p)=\text{exp}(-\sum_{i=1}^{S}p_{i}\text{ ln }p_{i}),$$

(4) $$D_{q=2}(p)=\frac{1}{\sum_{i=1}^{S}p_{i}^{2}}.$$

*q* denotes the order of the Hill-number, which is part of a general family that can be denoted as *D_q_*(**p**), and expresses the weight that is given to more abundant species. *D_q_*_=1_ is the equivalent of the exponential of the Shannon entropy, and *D_q_*_=2_ is the equivalent of the inverse Simpson index (50).

*β*-Diversity metrics quantify the difference in compositions between different communities. We quantify the dissimilarity between samples using the Bray-Curtis dissimilarity (51). If we let *BC_AB_* denote the dissimilarity between communities *A* and *B*, *BC_AB_* is calculated using the following equation:

(5) $$BC_{\mathrm{AB}}=\frac{\sum_{i=1}^{S}|p_{A,i}\;-\;p_{B,i}|}{\sum_{i=1}^{S}|p_{A,i}\;+\;p_{B,i}|}$$

### Predictive modeling.

FCM fingerprints can be used as input variables to train a machine learning model. We use Random Forest regression (52), an ensemble of decision trees, to predict α-diversity values, based on the *in silico* assembling strategy to estimate the structure of synthetic microbial communities (see below). A randomized grid search is performed to search for an optimal hyperparameter combination (53). This means that a number of random combinations of hyperparameter values were evaluated. The maximum number of variables that are considered at an individual split for a decision tree is randomly drawn from {1, …, *K*}, and the minimum number of samples for a specific leaf is randomly drawn between {1, …, 5}. One hundred different combinations were evaluated using 5-fold cross-validation, and predictions were reported for a separate test set.

### Data sets.

**(i) *In silico* synthetic bacterial communities.** Data from 20 individual bacterial strains, which were grown in the laboratory and measured by FCM, were collected from reference 10. In brief, individual bacterial cultures were sampled after 24 h of incubation and stained with SYBR green I, and two technical replicates per strain were measured on an Accuri C6 (BD Biosciences). Fluorescence was measured by the targeted detector (FL1, 530/30 nm) and three additional detectors, next to forward (FSC) and side (SSC) scatter. After background removal, additional automated denoising was performed using the FlowAI package (v1.4.4., default settings; target channel, FL1; changepoint detection, 150 [54]). A full experimental overview can be found in reference 10. The lowest number of cells collected after background removal amounted to 13,166 cells. The data are available via FlowRepository (accession ID: FR-FCM-ZZSH).

**(ii) Cooling water microbiome.** Data were used as presented in reference 13. Samples were collected from the cooling water of a discontinuously operated research nuclear reactor. This reactor underwent four phases: control, startup, operational, and shutdown. Samples were taken from two surveys in time (surveys I and II) and analyzed via FCM and 16S rRNA gene amplicon sequencing (*n*_survey I_ = 36 and *n*_survey II_ = 31). The procedure and data preprocessing are described in reference 13. In brief, samples were stained with SYBR green I, and three technical replicates were analyzed using an Accuri C6 (BD Biosciences). Fluorescence was measured by the targeted detector (FL1, 530/30 nm) and three additional detectors, next to forward (FSC) and side (SSC) scatter. The data are available via FlowRepository (accession ID: FR-FCM-ZZNA). The lowest number of cells collected after background removal amounted to 10,565 cells. Taxonomic identification of microbial communities was done at the operational taxonomic unit (OTU) level at 97% similarity after preprocessing. All the samples were subsampled down to the minimum sequencing depth and normalized afterward. Sequences are available from the NCBI Sequence Read Archive (SRA) under accession ID SRP066190.

**(iii) Freshwater lake microbiome.** Data were collected as presented in reference 55. A total of 173 samples, from three types of freshwater lake systems, were analyzed through 16S rRNA gene amplicon sequencing and FCM. Samples originated from three different freshwater lake systems: (i) 49 samples from Lake Michigan (2013 and 2015), (ii) 62 samples from Muskegon Lake (2013 to 2015; one of Lake Michigan’s estuaries); and (iii) 62 samples from 12 inland lakes in southeastern Michigan (2014 to 2015). Field sampling, DNA extraction, DNA sequencing, and processing are described in reference 56. Fastq files were submitted to NCBI SRA under BioProject accession numbers PRJNA412984 and PRJNA414423. Taxonomic identification of microbial communities was done for each of the three lake data sets separately and treated with a minimum OTU abundance threshold cutoff of one sequence in 3% of the samples. Sequences were clustered into OTUs at 97% similarity. Each of the three data sets was rarefied to an even sequencing depth, which was 4,491 sequences for Muskegon Lake samples, 5,724 sequences for the Lake Michigan samples, and 9,037 sequences for the inland lake samples. The relative abundance at the OTU level was calculated by taking the count value and dividing it by the sequencing depth of the sample. Flow cytometry procedures are described in reference 25. In brief, samples were stained with SYBR green I, and three technical replicates were measured on an Accuri C6 (BD Biosciences). Fluorescence was measured by the targeted detector (FL1, 530/30 nm) and three additional detectors, next to forward (FSC) and side (SSC) scatter. FCM data are available via FlowRepository (accession IDs: FR-FCM-ZY9J and FR-FCM-ZYZN). The lowest number of cells collected after denoising amounted to 2,342 cells.

### Method evaluation.

Our proposed fingerprinting approach based on GMMs was compared to a generic fixed binning approach, which we have called PhenoGrid. In brief, we implemented a binning grid of *L *= 128 × 128 for each bivariate FCM channel combination, after which cell fractions per bin were determined. The resulting cell fractions were next vectorized, concatenated, and normalized. Both PhenoGMM and PhenoGrid result in multiple variables that describe relative cell counts, either per mixture or per bin. These methods were evaluated to estimate the structure of both synthetic and natural communities.

**(i) α-Diversity estimations of *in silico* synthetic microbial communities.** In the first setup, we assessed how well PhenoGMM was able to capture variations in the structure of synthetic microbial communities. To do so, we first performed an *in silico* community assembly strategy. In other words, cytometric characterizations of individual bacterial strains were artificially aggregated according to simulated compositions (10). These compositions were determined according to the following strategy:

1. Sample at random a number *S*′*_i_* that represents the number of different members that will constitute the microbial community *i*. *S*′*_i_* lies between two and 20 (the total number of strains that are available).
2. The Dirichlet distribution can be used to model the joint distribution of individual fractions of multiple species (57). We applied the Dirichlet distribution to randomly simulate the composition of microbial community *i*. The evenness of the composition depends on the concentration parameter *a* of the Dirichlet distribution, which determines how evenly the weight will be spread over multiple community members. If *a* is low, only a few members will make up a large part of the community (low evenness). If *a* is high, the fraction of each member contributing to the community composition will be close to equal (high evenness).

Four hundred community compositions (300 training and 100 test communities) were simulated for three different values of *a *= 0.1, 1, and 10. The simulated compositions were visualized using Lorenz curves (see Fig. S1 in the supplemental material). Next, *in silico* synthetic bacterial communities were assembled by aggregating the cytometric characterizations of individual bacterial strains according to these simulated compositions. Diversity values could be calculated with high accuracy based on the simulated compositions by calculating the Hill numbers for *q *= 0, 1, and 2 and Bray-Curtis dissimilarities for these compositions. These were then correlated with the relative cell abundances that resulted from PhenoGMM and PhenoGrid. The strength of the correlation was assessed using Kendall’s τ*_B_* and a Mantel test. The Random Forest prediction experiment was additionally evaluated using the *R*_2_ (see below).

**(ii) α-Diversity estimations of natural microbial communities.** Cytometric diversity estimations for natural communities (i.e., the cooling water and freshwater lake microbiome) were evaluated in a different way. To benchmark PhenoGMM, these values were correlated with α- and *β*-diversity values based on 16S rRNA gene amplicon sequencing, motivated by previous reported correlations between the cytometric and taxonomic diversity (13, 25). The strength of the correlation was assessed using Kendall’s τ*_B_* and a Mantel test (see below).

### (iii) Performance evaluation.

• α-Diversity estimations were quantified by calculating Kendall’s rank correlation coefficient *τ_B_* between the true and estimated values. The *τ_B_* implementation, which is able to deal with ties, was calculated as follows:
  (6) $$\tau_{B}=\frac{N_{c}\;-\;N_{d}}{\sqrt{\left(N_{c}\;+\;N_{d}\;+\;N_{t})\times (N_{c}\;+\;N_{d}\;+\;N_{u}\right)}}.$$
  *N_c_* denotes the number of concordant pairs between true and predicted values, *N_d_* the number of discordant pairs, *N_t_* the number of ties in the true values, and *N_u_* the number of ties in the predicted values. Values range from −1 (perfect negative association) to +1 (perfect positive association), and a value of 0 indicates the absence of an association. This was done using the kendalltau() function in Scipy (v1.0.0) (59).
• Random Forest predictions were evaluated by calculating the *R*^2^ between true (**y** = {*y*_1_, …, *y_n_*}) and predicted (**ŷ** = {*ŷ*_1_, …, *ŷ_n_*}) values:
  (7) $$R^{2}(y,\hat{y})=1-\frac{\sum_{i=0}^{n-1}{\left(y_{i}\;-\;{\hat{y}}_{i}\right)}^{2}}{\sum_{i=0}^{n-1}{\left(y_{i}\;-\;\bar{y}\right)}^{2}},$$
  in which *y* denotes the average value of **y**. If *R*^2^ = 1, predictions are correctly estimated. If *R*_2_ < 0, predictions are worse than random guessing. The r2_score()-function from the scikit-learn machine learning library was used (46).
• *β*-Diversity estimations were evaluated by calculating the correlation between Bray-Curtis dissimilarity matrices (*BC*) based on FCM and 16S rRNA gene sequencing data using a Mantel test (58). This test assesses the alternative hypothesis that the distances between samples based on cytometry data are linearly correlated with those based on 16S rRNA gene sequencing data. It makes use of the cross-product term *Z_M_* across the two matrices for each element *ij*:
  (8) $$Z_{M}=\sum_{i=1}^{n}\sum_{j=1}^{n}BC_{\mathrm{ij}}^{\text{FCM}}\times BC_{\mathrm{ij}}^{\text{16S}}.$$
  The test statistic *Z_M_* is normalized and then compared to a null distribution, based on 1,000 permutations.

### Code and data availability.

All code and data supporting this article are freely available on GitHub at https://github.com/prubbens/PhenoGMM. The functionality of PhenoGMM has been incorporated in the R package PhenoFlow: https://github.com/CMET-UGent/Phenoflow_package. Raw flow cytometry data are freely available on FlowRepository (accession numbers FR-FCM-ZZSH, FR-FCM-ZZNA, FR-FCM-ZY9J, and FR-FCM-ZYZN). Raw sequences are available via the NCBI Sequence Read Archive (accession numbers SRP066190, PRJNA412984, and PRJNA414423).
