# Supplementary figures and images for: PhenoGMM: Gaussian Mixture Modeling of Cytometry Data Quantifies Changes in Microbial Community Structure
Source: mSphere. 2021 Feb 3;6(1):e00530-20. doi: 10.1128/mSphere.00530-20 (PMC7860985; doi:10.1128/mSphere.00530-20)

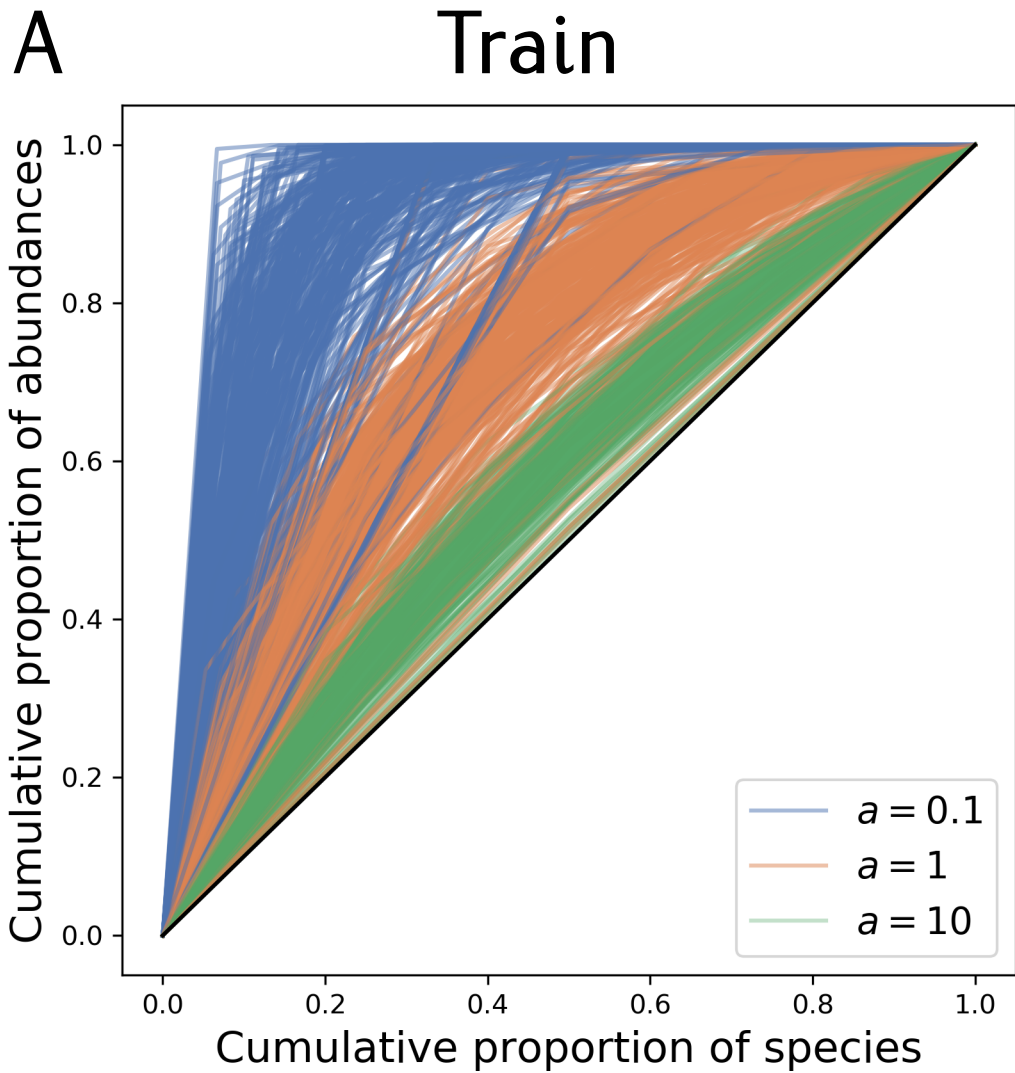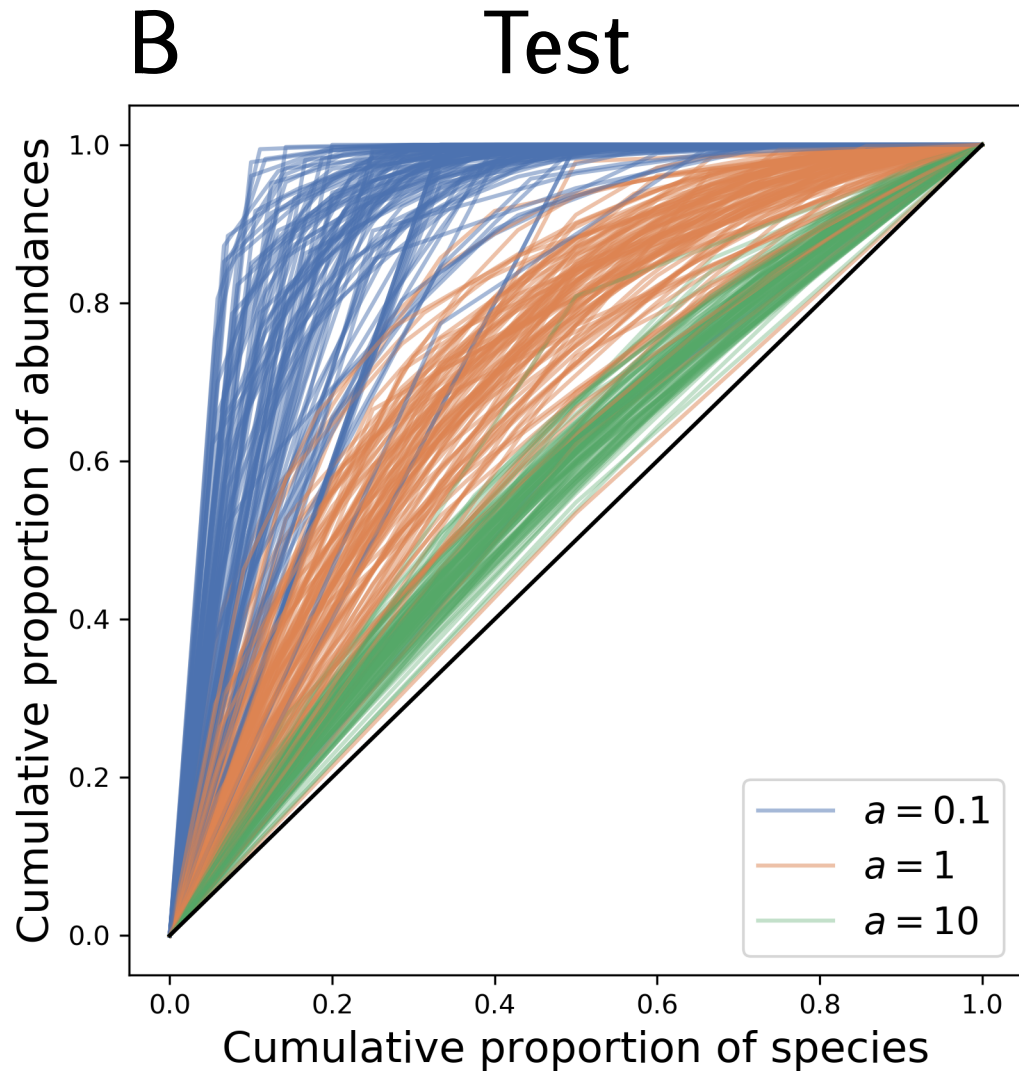

Supplement: FIG S1 [file mSphere.00530-20-sf001.pdf]

**A**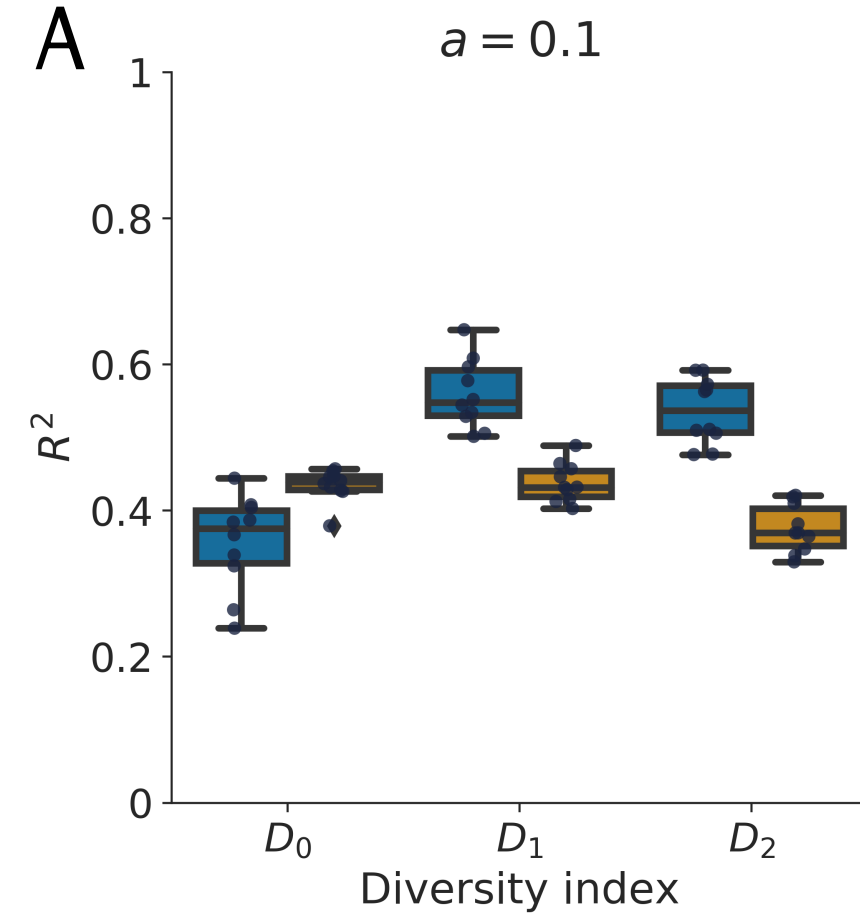**B**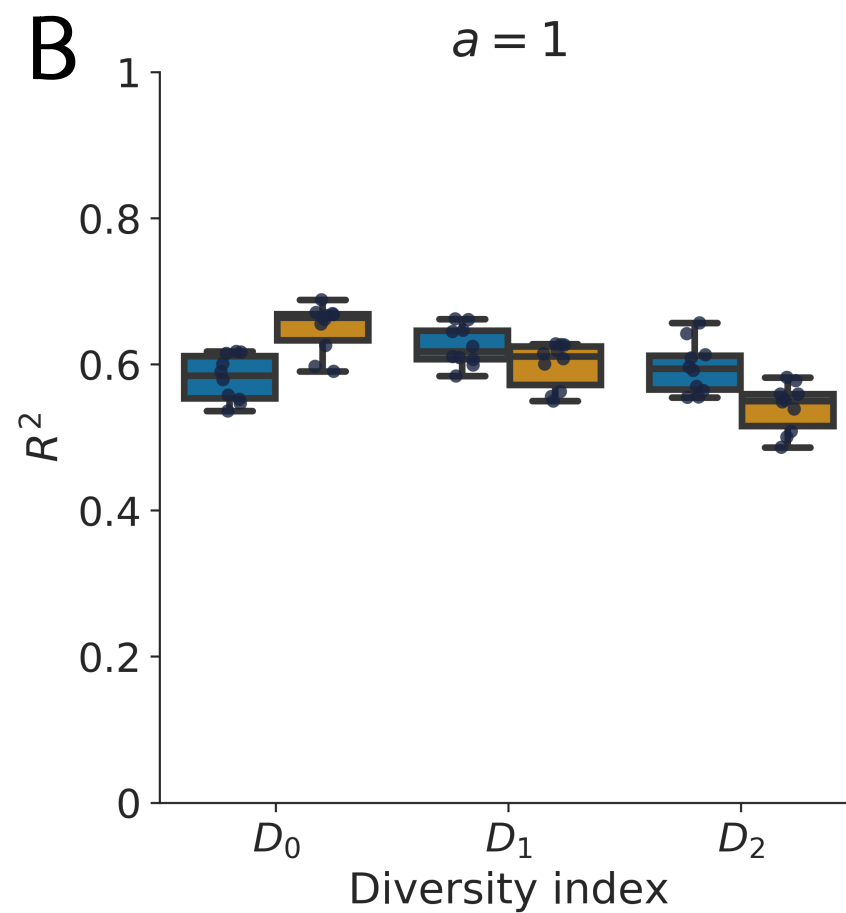**C**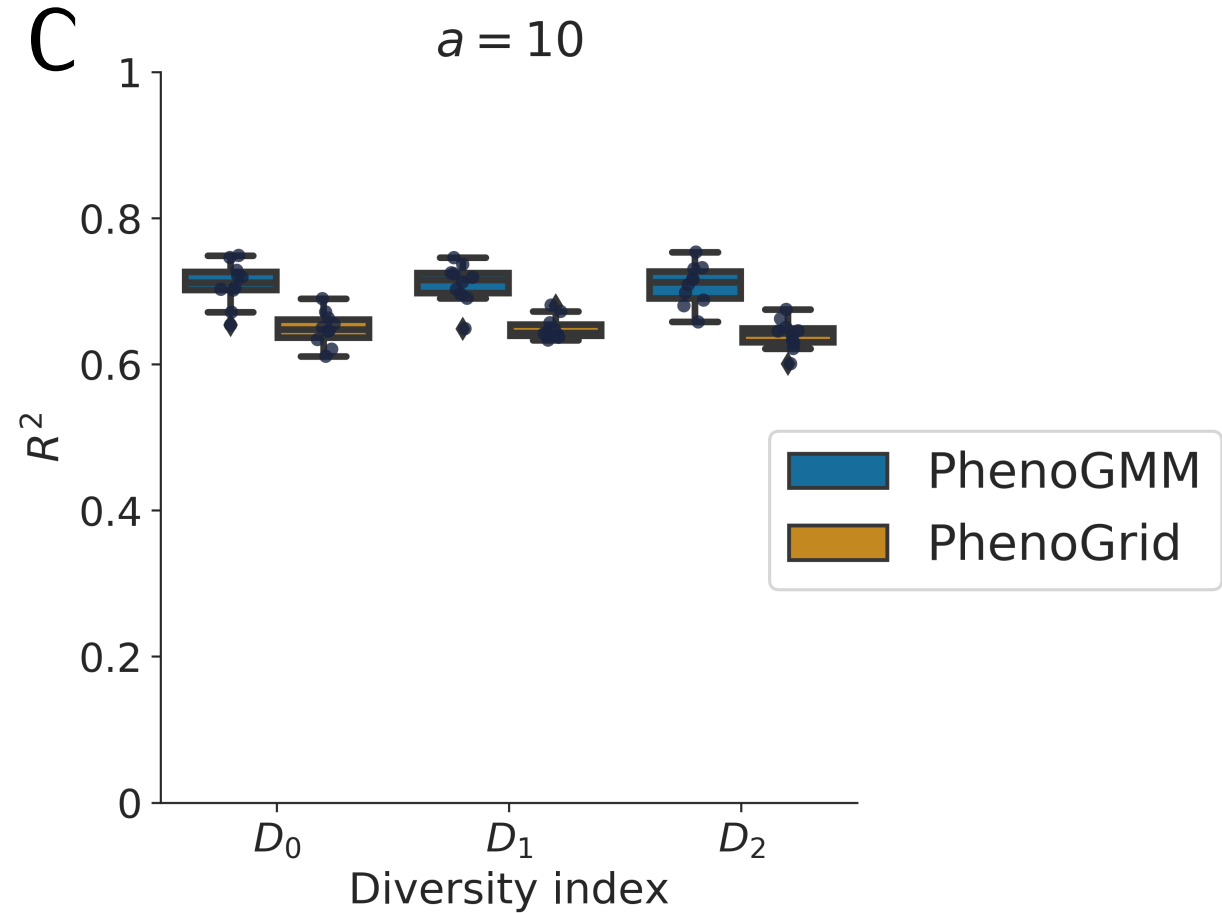

Supplement: FIG S2 [file mSphere.00530-20-sf002.pdf]

# A: Unsupervised

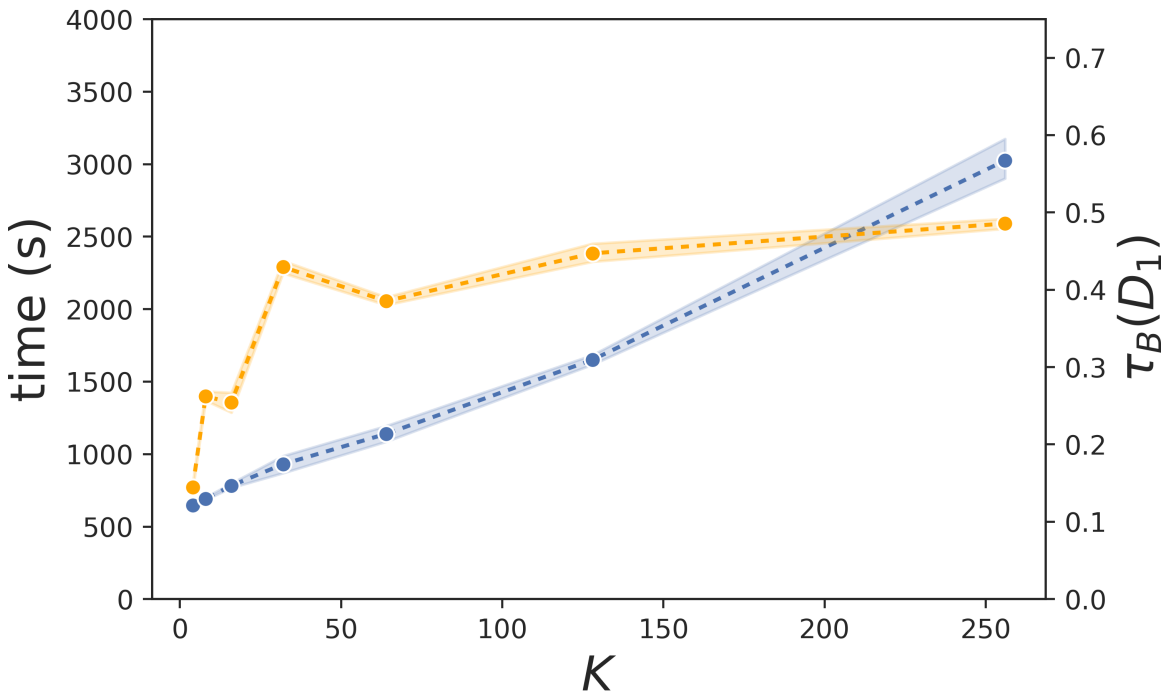

# B: Supervised

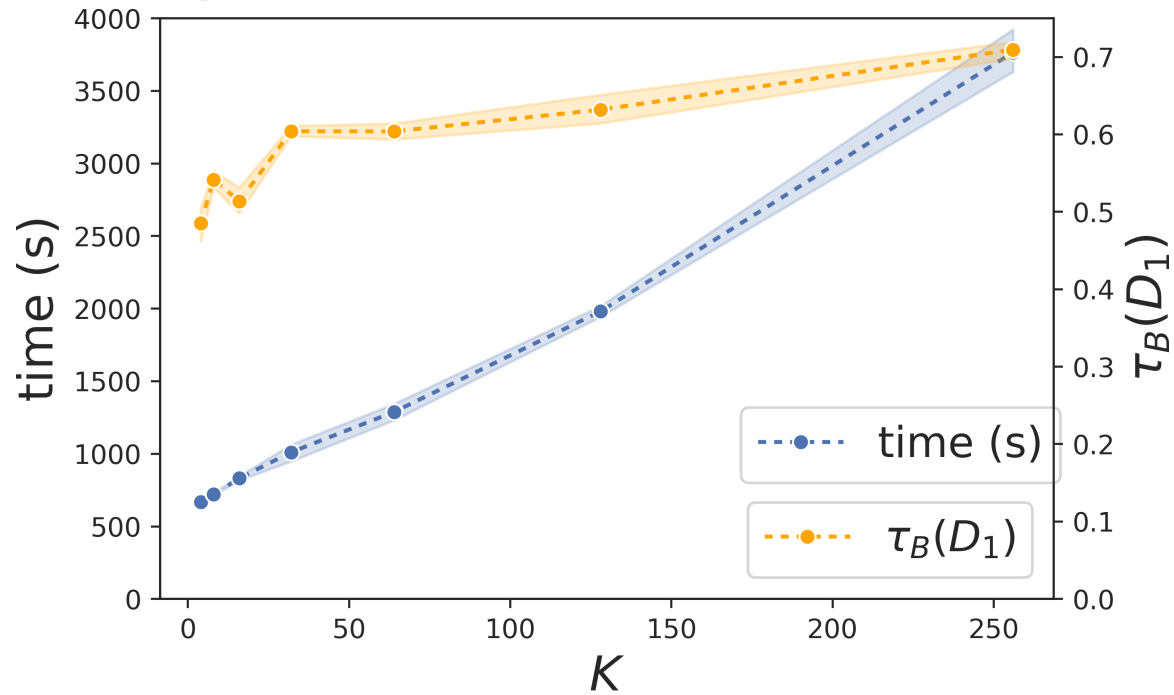

Supplement: FIG S3 [file mSphere.00530-20-sf003.pdf]

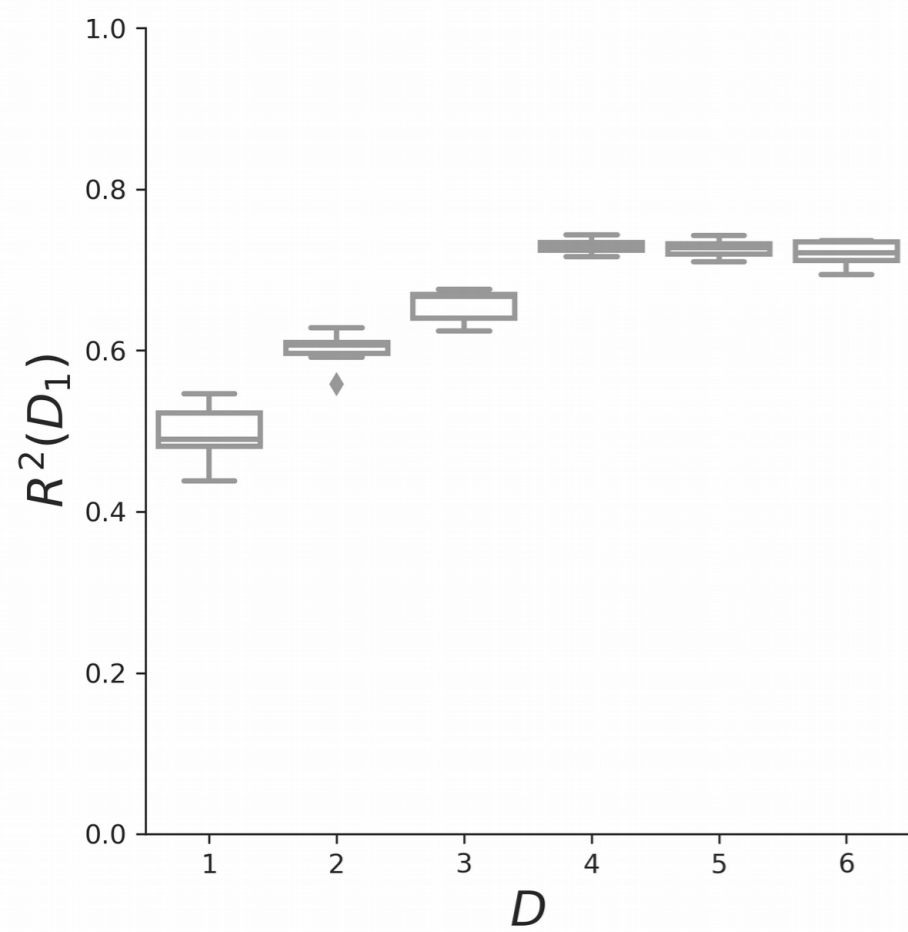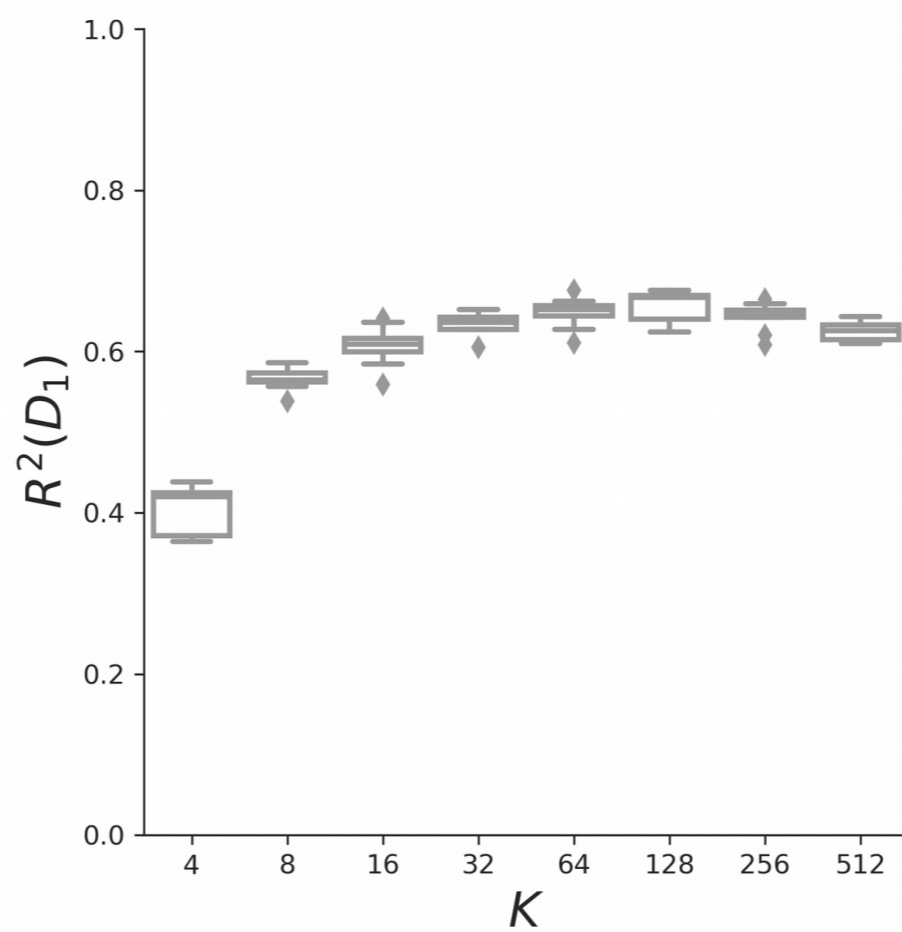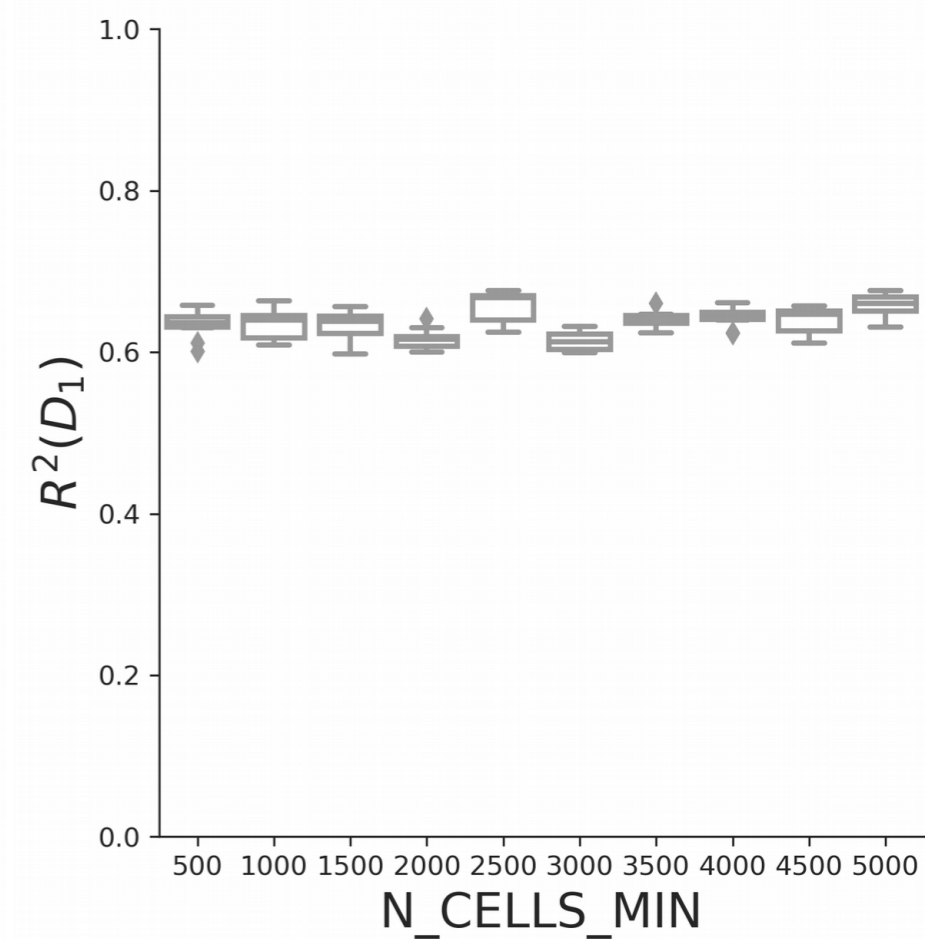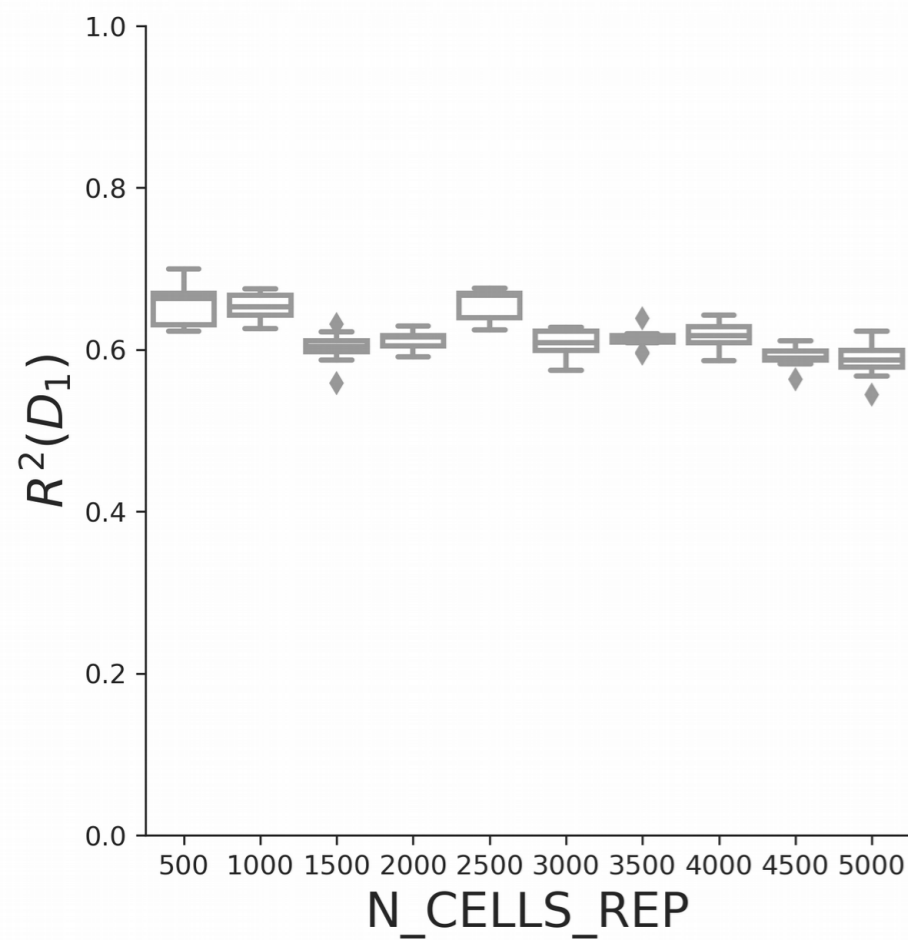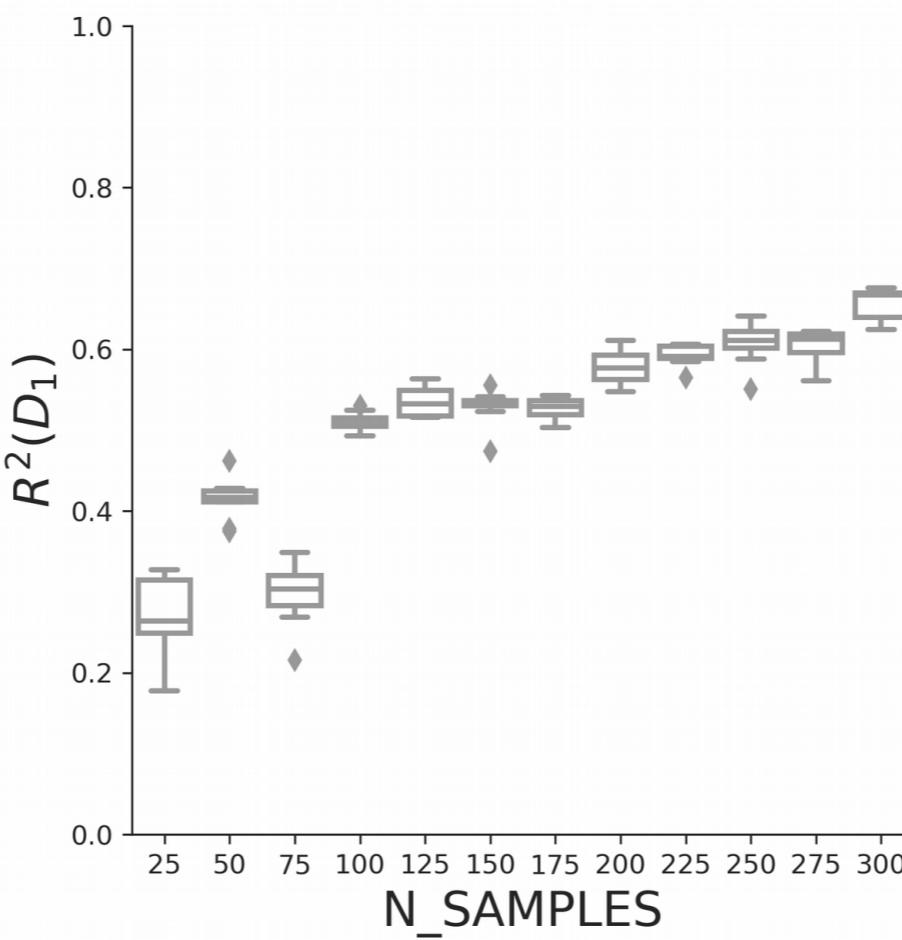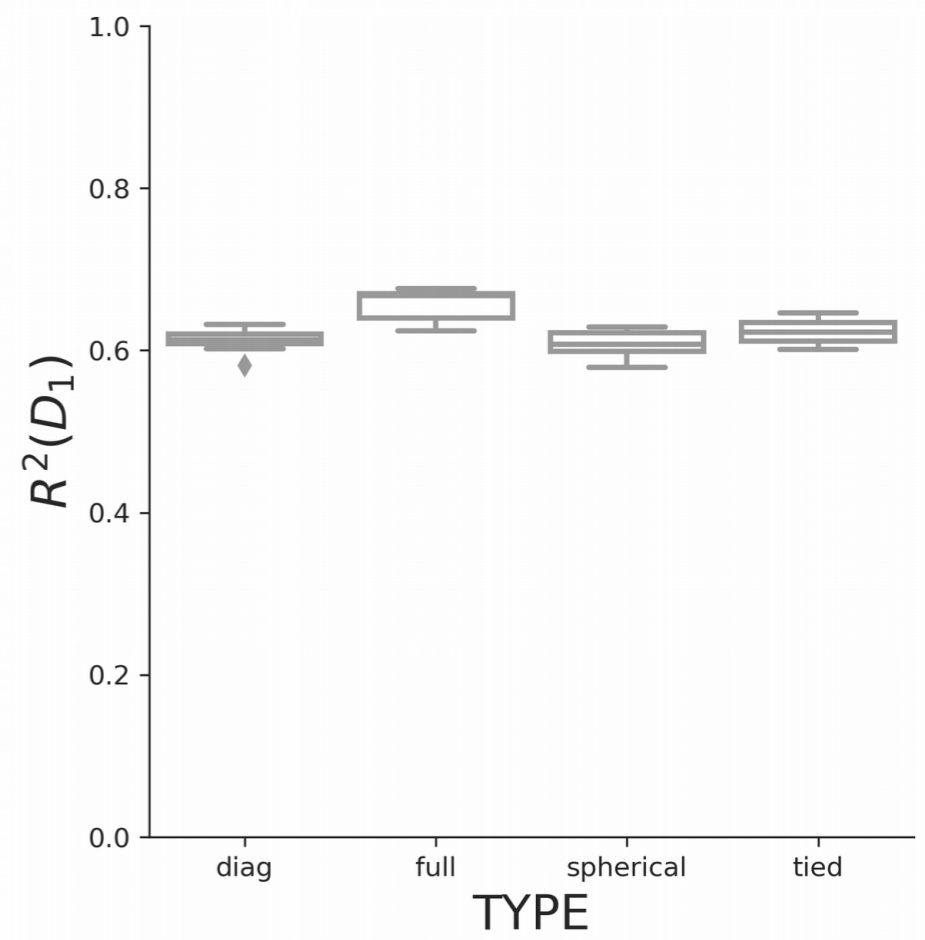

Supplement: FIG S4 [file mSphere.00530-20-sf004.pdf]
